# Scientific Items

**Published:** 1882-12-20

**Authors:** 


### SCIENTIFIC ITEMS.

**Gun-Powder as a Motive Power.**—A patent has just been taken out in Germany for an engine, the piston of which is driven backward and forward by small charges of gun-powder supplied at each end by an automatic arrangement. The ignition is effected by the motion of the piston, which draws in a flame of gas or spirit, the access being regulated by side valves, which also open outlets for the escape of the gases of combustion.

A SERIES of magnetic observations, which are to extend over a space of fourteen months, have recently been commenced at Goettingen, Germany. They will take place under the supervision of the Professor of the University, on the 1st and 15th of every month, at the same hours as those performed by the international expeditions sent out to the North and South Poles. Their principal object is to ascertain the magnetic condition of the earth. Experiments will also be made with respect to magnetic intensity in the garden of the observatory, in a pavilion built up of wood and brick only, iron being omitted on account of the disturbing influences which it would exert, rendering the observations practically valueless.—*Mechanical News.*

**The Tails of Comets.**—Professor Ennis, of the Naval Observatory at Washington, believes that the tails of comets are electric light. "If these tails had any substance," he argues, "the laws of motion are constantly violated by them. The great comet of 1843 went so near the sun that it passed from one side to the other in a few hours. Its immense tail, 100,000,000 miles long, was shifted completely, so that it pointed directly in an opposite direction. Could that be so if it were composed of any substance? Could a comet swing 100,000,000 miles of tail around so quick as that? The electricity is generated by evaporation. As the comets approach the sun, the heat becomes more intense, the evaporation and accumulation of electricity more rapid, the repulsive force greater, and the tails longer. Sometimes the material becomes completely evaporated. Then the comet has no tail."—*Literary Microcosm.*

THE new metal, of which it is proposed to construct pipes in which to lay telegraph wires under ground, is described as very light—only about one-sixth the weight of iron—and, being composed almost entirely of pure carbon, is indestructible, whether in the air or under ground. It does not rust or change by exposure, and is not affected by heat or frost. The most important characteristic claimed for it, however, in connection with underground wires, is its being a perfect insulator. The pipes of the metal need not, it is stated, be buried very deep in the ground, as they may be of a semi-elastic character, adjusting themselves to the slight upheaval and depression of the ground through the action of frost.—*Literary Microcosm.*

THE lake that has the highest elevation of any in the world is Green Lake, in Colorado. Its surface is 10,252 feet above the level of the sea. Pine forests surround it, and eternal snows deck the neighboring mountain-tops. One of these, Gray's Peak, has an altitude of 14,341 feet. The water of Green Lake is as clear as crystal, and large masses of rock and a petrified forest are distinctly visible at the bottom. The branches of the trees are of dazzling whiteness, as though cut in marble. Salmon and trout swim among them. In places the lake is 200 feet deep.—*Literary Microcosm.*

**The Microscopists at Dinner.**—The New York *Times* indulges in a little good-natured fun at the prevalent mania for finding the source of all evils in microscopic organisms. It reports an imaginary meeting of microscopists, and the following is an extract from the "proceedings:"

When the microscopists sat down to dinner each one produced his compound oscillating microscope, and carefully examined every article of food. Excited shouts went up as new discoveries of metallic, vegetable and sausage substances were discovered in the soup. An examination made of the water resulted in the discovery of such an enormous quantity of infusoria. mammalia and pachydermata that the microscopists unanimously refused to drink it. During the progress of the meal much enthusiasm was aroused by the announcement of Professor White that he had discovered a trace of hairpin in the beefsteak, thus upsetting the theory that the beefsteak of American hotels is a chemically pure carburet of sole-leather; and, at a later hour, Professor Black's assertion—based on a thorough microscopic examination—that he had discovered whortleberries in the whortleberry pudding, and wine in the wine sauce, led to a heated discussion, in the course of which thirty-eight microscopists declared that Professor Black was an ignorant and unprincipled pretender, and eleven others maintained that the professor was acting in good faith, and that his discoveries could be accounted for on the theory that the waiter had given him, by mistake, a piece of whortleberry pudding made expressly for the landlord's private table.—*Journal of Chemistry.*

**Chemical Deep-Sea Soundings.**—Sir William Thompson's apparatus for deep-sea sounding consists of a glass tube filled with air, sealed at the top, but open at the bottom, and prepared inside with red prussiate of potassa. It is placed in a brass tube, closed at the bottom, but open at the top. The brass tube is partially filled with ferrous sulphate, which, when coming into contact with the red prussiate inside the glass tube, forms prussian blue. When the instrument is thrown into the sea, the pressure of the water compresses the air, forcing the ferrous solution up the glass tube, according to the depth to which it descends. On measuring the length of the blue stain the depth of the sounding is ascertained.—*Drug. Cir.*

